# Predictors of Quitting Smoking in Cardiac Rehabilitation

**DOI:** 10.3390/jcm9082612

**Published:** 2020-08-12

**Authors:** Ahmad Salman, Patrick Doherty

**Affiliations:** Department of Health Sciences, University of York, York YO10 5DD, UK; patrick.doherty@york.ac.uk

**Keywords:** cardiac rehabilitation, smoking, quitting

## Abstract

Quitting smoking and participation in cardiac rehabilitation (CR) are effective strategies in reducing morbidity and mortality. However, little is known about the predictors of quitting smoking in those who attend CR. This study aimed to determine the sociodemographic and clinical factors associated with the likelihood of CR attendees who are quitting smoking. Data from the UK National Audit of Cardiac Rehabilitation (NACR) database, between April 2013 and March 2016, were used. Smoking status is categorized as smokers and quitters, assessed by patient self-report. The study used patient demographics, cardiovascular risk factors, comorbidities, and physical and psychosocial health measures. Binary logistic regression was performed to identify the predictors of quitting smoking among CR attendees. Of the 3290 patients who started CR and were entered into the NACR database, 2052 were continued smokers (mean age 58.59 ± 10.49 years, 73.6% men) and 1238 were quitters (mean age 57.63 ± 10.36 years, 75.8% men). The median duration of CR was 9 weeks. Compared to smokers, the quitters were younger, weighed more, were less anxious and depressed, and were more likely to be employed. Single patients had 0.60 times lower odds (95% CI 0.43 to 0.82) of quitting smoking than patients with partners, and low-risk patients had 1.71 times higher odds (95% CI 1.12 to 2.62) of quitting smoking than high-risk patients. Increasing number of comorbidities and depression scores were associated with decreasing likelihood of quitting. This study highlights the routine factors that determine smoking cessation outcomes, which could inform the delivery of CR to better help patients to quit smoking.

## 1. Introduction

Smoking is a major risk factor for cardiovascular disease (CVD) and one of the biggest threats that the world has ever faced, being the cause of death of more than 8 million people per year [1]. The recent highly comprehensive meta-analysis of the link between smoking and CVD used data from 25 prospective cohorts in the Consortium on Health and Ageing: Network of Cohorts in Europe and the United States (CHANCES). It found that smoking is a strong independent risk factor for CVD and mortality in people aged ≥60 years [2]. This analysis found that smokers had a two-fold higher risk of cardiovascular mortality as compared to non-smokers, smoking advanced the risk of death from CVD by more than five years, smoking cessation in older adults is still beneficial, and the increased excess risk among quitters declined with time after smoking cessation [2].

A meta-analysis of 12 cohort studies estimated that mortality in patients who continue to smoke after myocardial infarction (MI) is 20% and suggested that smoking cessation is associated with a significant decrease in mortality [3]. A retrospective analysis of data from an American study showed that people who continued to smoke after percutaneous coronary revascularization had a 76% increased risk of death after an average of 4.5 years of follow-up, as compared to non-smokers, and a 44% higher risk of death compared to those who quit smoking [4].

Stronger evidence comes from a 15-year follow-up of Dutch patients who underwent coronary bypass surgery [5]. Patients who were smoking 1 year after surgery had a risk of subsequent myocardial infraction and reoperation more than two times higher than that of patients who had quit smoking since surgery [5]. Patients who were still smoking at 5 years after surgery had an even higher risk of MI and reoperation and a significantly increased risk of angina pectoris, as compared to patients who stopped smoking after surgery and patients who never smoked. Moreover, risks of MI were similar among non-smokers and those who were successful in quitting after surgery [5].

Smoking is a major preventable risk factor for the development of non-communicable diseases, including cardiovascular, respiratory diseases, and cancers [6]. Quitting smoking is the most cost-effective strategy for CVD prevention [7]. A systematic review of 20 prospective cohort studies showed that quitting smoking is associated with a 36% reduction in the risk of all-cause mortality for patients with coronary heart disease who quit, compared to those who continued smoking [8]. International guidelines recommend that CVD prevention should be delivered in patients at moderate to high risk of CVD and patients with established CVD, by tackling smoking as a risk factor. Furthermore, they considered quitting smoking as an important target in both primary and secondary prevention of CVD [7,9,10,11]. Adopting healthy behaviors such as quitting smoking is the cornerstone of effective preventive and control efforts of CVD. It is postulated that elimination of health-risk behaviors could prevent at least 80% of cases of CVD and 40% of cancers [9,10,11,12,13].

Cardiac rehabilitation (CR), a comprehensive intervention offered to patients with CVD, is a structured multidisciplinary intervention designed to target risk factors and psychosocial wellbeing and supports smoking cessation in attempts to improve or maintain psychosocial wellbeing [7,9,14]. Participation in CR is associated with reduced cardiovascular mortality and hospital readmission among other benefits [15]. Accordingly, patients in the United Kingdom (UK) and several other countries have access to secondary prevention CR programs. Average uptake to UK CR programs reached 50%, which is considered the highest uptake figures globally [16]. Across the UK, CR is delivered in accordance with the British Association for Cardiovascular Prevention and Rehabilitation (BACPR) standards, which aim to reduce cardiovascular risk and promote quality of life through coordinated core components of CVD prevention and rehabilitation [9]. Through the lifestyle risk factor management of its core component, the BACPR recommends supporting people with smoking cessation and relapse prevention [9]. The average proportion of patients who entered CR as non-smokers in the UK was 94% [16]. CR is associated with an average increase in smoking cessation of one percent (1.1%) [17].

Although extensive research was carried out on CR, little is known about the factors that are associated with patients quitting smoking among CR participants. There is substantial evidence that the demographic characteristics associated with quitting smoking include gender [18,19], age [18,19,20,21], and marital status [22,23,24,25,26]. Smoking-related variables include tobacco dependence, the baseline number of cigarettes smoked per day, the number of previous quit attempts, motivation to quit, and the proportion of smokers in the household, all of which might influence the success of quitting smoking [18,22,27,28,29]. Predictors of successful quitting include lower levels of depression [29,30]. Better designed observational studies are needed to determine the factors that are associated with successfully quitting smoking in CR attendees. To date, research on the determinants of the likelihood of quitting smoking among CR attendees is limited. A more thorough investigation is required to identify the predictors of quitting smoking in CR, thus informing tailored interventions to increase quitting smoking. This study investigates the sociodemographic and clinical factors associated with the likelihood of quitting smoking among CR attendees.

## 2. Methods

### 2.1. Data Source

The analyses were conducted using individual patient data collected electronically in the National Audit of Cardiac Rehabilitation (NACR). The NACR is a web-based registry of CR in the UK, funded by the British Heart Foundation. Practitioners involved in CR delivery electronically enter data on eligible patients who are referred for CR into the individual patient dataset, according to a data dictionary (www.cardiacrehabilitation.org.uk/nacr/downloads.htm), and the data quality is checked by a member of the NACR team. The audit is voluntary, supports direct entry of data within a secure online system, and collects local program-level data for those who are referred to and undergo CR. It includes details of a patient’s initiating event, treatment type, risk factors, drugs, patient demographics, and post-CR clinical outcomes. The NACR has approval to collect anonymized patient data for a range of clinical variables, without explicit consent from individual patients, for the purposes of audit and research under Section 251 of the NHS Act 2006 [16]. Approval is reviewed annually by NHS Digital. Separate ethical approval was not required as a part of this research in addition to the e-survey project, which is also an NACR audit process. This observational study was reported, following the guidelines of the Strengthening the Reporting of Observational Studies in Epidemiology (STROBE) guidelines [31] (Supplementary Materials: Table S1).

### 2.2. Participants

The research cohort included data from patients added to the NACR database between 1 April 2013 and 31 March 2016, which were validated and extracted retrospectively. The analysis included data on sociodemographic and clinical characteristics for patients who started CR and had a smoking status assessment at baseline (pre-CR) and follow-up (post-CR). There were no exclusion criteria.

### 2.3. Smoking Outcome Measures

Smoking status in the NACR database is recorded with information obtained by patient self-report questionnaires [16]. Patients are categorized according to smoking status pre- and post-CR to one of the following statuses:

- Never smoked
- Ex-smoker
- Stopped smoking since event
- Currently smoking.

From the smoking status record, pre- and post- for each CR patient, patients were defined, for the purposes of this research, as:

- continued smokers, if they were current smokers in both the pre- and post-CR assessments.
- ex-smokers, if they were current smokers at the pre-CR assessment and stopping smoking status at the post-CR assessment.

### 2.4. Baseline Characteristics

Numerous past studies used a variety of baseline characteristics to assess the difference between continued smokers and quitters [18,19,20,21,26,32,33,34]. The present study used a variety of different patient variables collected by the NACR primary dataset, including anthropometric data, sociodemographic data, comorbidities, cardiovascular risk factors, and physical and psychosocial health measures. Anthropometric measurements that are available in the NACR database include weight (kg), height (m), body mass index (BMI) (kg/m^2^), and waist (cm). The sociodemographic data collected includes age, gender, marital status, work status, and ethnicity.

Comorbidities are any of the 19 commonly associated conditions that the patients who undergo CR have, such as angina, diabetes, and cancer. These comorbidities are routinely collected by the NACR and reported in the national statistical report [16]. Risk stratification is a multifactorial measure used to establish prognosis of future major cardiac events or exercise complications, using all relevant patient information, e.g., left ventricular ejection fraction, history of arrhythmia, symptoms, and functional capacity [9,35,36,37]. Mortality risk within the first year is 2% for an individual assessed as low risk, 10–25% for an individual assessed as moderate risk, and >25% for those assessed as high risk [9,35,36,37]. If blood pressure (BP) is ≥140/90 mmHg, a patient is considered to have hypertension [38]. Alcohol consumption status in the NACR database is recorded using information obtained from patient self-report questionnaires about weekly alcohol consumption [39]. Where a weekly unit amount was recorded, the patient was allocated to the relevant category, based on their gender and the recommended weekly units. The National Institute for Health and Care Excellence (NICE) guidelines recommend that men should not regularly drink more than 21 units of alcohol per week and women should not regularly drink more than 14 units of alcohol per week [40].

To evaluate the role of social deprivation, the study included the English Index of Multiple Deprivation (IMD) Lower Layer Super Output Areas (LSOA) deprivation deciles, which linked to the NACR [41]. These deciles are ranked from 1 to 10, where 1 means that that the LSOA is among the most deprived 10% nationally and 10 represents the least deprived 10%. Self-reported moderate physical activity (150 min/week; yes/no) and self-reported vigorous physical activity (75 min/week; yes/no)) conforming to the Department of Health guidelines for 19–64 and 65+ age groups [42]. Psychosocial health status was assessed on the Hospital Anxiety and Depression Scale (HADS) scores, a reliable and well-validated scale, with higher scores representing worse symptoms [43].

### 2.5. Statistical Analysis

All analyses were performed in the IBM Statistical Package for Social Sciences (SPSS) software Version 26 (IBM, New York, NY, USA). *p* < 0.05 was considered to be statistically significant.

Smoking status was valued as 1 for quitters and 0 for smokers. Frequency tables were generated to categorize CR patients as smokers and quitters, according to their recorded pre- and post-CR smoking status. Analyses were conducted using all available data from CR attendees to minimize selection bias. Continuous variables are shown as mean (standard deviation (SD)) and categorical variables as frequencies (percentage).

Descriptive statistics were used to describe and compare a variety of sociodemographic and baseline characteristics between smokers and quitters, among CR attendees in the UK. Both continuous and categorical variables were used, depending on the method of data collection in the NACR. Differences in baseline characteristics were then compared using independent-sample *t*-test for continuous variables or chi-square test for categorical variables. Cohen’s d test was used as a measure of the effect size to indicate the mean difference between two groups in standard deviation units [44]. In addition, a χ^2^ test for association was conducted between baseline sociodemographic and clinical characteristics of smokers and quitters participating in CR [44]. Phi and Cramér’s V tests are a measure of the effect size or strength of association of a nominal by nominal relationship [44]. Binary logistic regression was used to predict the probability of quitting smoking among CR attendees. Variables were considered in the equation for the binary logistic analysis, based on the extent of association between smokers and quitters [45].

## 3. Results

### 3.1. Cohort Characteristics

The NACR cohort included 130,961 patients who started CR during the research period. A total of 48,820 (37.3%) had a missing smoking status. Overall, 91.4% of patients who started CR and had a smoking status recorded were classified as non-smokers (31,832 had never smoked; 35,417 were ex-smokers; 7,808 had stopped smoking since event).

Of the 49,725 patients who had their smoking status recorded pre- and post-CR, 46,435 (93.4%) were classified as non-smokers (mean age 65.72 ± 11.08 years, 74.7% male), 2052 (4.1%) as continued smokers (mean age 58.59 ± 10.49 years, 73.6% male), and 1238 (2.5%) as ex-smokers (mean age 57.63 ± 10.36 years, 75.8% male). The median duration of CR was 9 weeks. For the purposes of this research, patients were categorized as either continued smokers or quitters (Table 1).

### 3.2. Baseline Characteristics

The mean baseline characteristics of smokers and quitters are summarized in Table 2 and Table 3.

An independent-samples *t*-test was run to determine whether sociodemographic and clinical characteristics differed between smokers and quitters (Table 2).

A χ^2^ test for association was conducted between the baseline sociodemographic and clinical characteristics and the two smoking categories—smokers and quitters participating in CR (Table 3).

The following characteristics were considered in the final model to identify CR attendees who quit smoking:

- Age
- Marital status
- Employment status
- Cardiovascular risk
- Comorbidities
- Weight
- Hospital anxiety and depression scale (HADS)
- Anxiety score
- Depression score

A binary logistic regression was performed to ascertain the effects of the baseline characteristics on the likelihood that CR attendees quit smoking (Table 4).

The logistic regression model was statistically significant (χ^2^(10) = 59.32, *p* < 0.0001), explained 9.3% (Nagelkerke R^2^) of variance in smoking status, and correctly classified 64.7% of cases. Sensitivity was 25.5%, specificity was 87.6%, positive predictive value was 54.5%, and negative predictive value was 66.9%. The Hosmer–Lemeshow test in the final model was not statistically significant (*p* = 1.00), indicating that the model was an appropriate fit.

Only four predictor variables were statistically significant—marital status, cardiovascular risk, comorbidities, and HADS depression score (Table 4). Single patients had 0.60 times lower odds (95% CI 0.43 to 0.82) of quitting smoking than patients with partners, and low-risk patients had 1.71 times higher odds (95% CI 1.12 to 2.62) of quitting smoking than high-risk patients. Increasing number of comorbidities and HADS depression score was associated with decreasing likelihood of quitting.

## 4. Discussion

This retrospective secondary analysis of data from the NACR found that patients with high cardiovascular risk, multiple comorbidities, no partner(s), and more severe depression were less likely to quit smoking during CR. Age, comorbidities, cardiovascular risk, marital status, work status, weight, HADS anxiety and depression scores expressed statistically significant differences between continued smokers and quitters. Compared to continued smokers, quitters were younger, weighed more, had fewer comorbidities, lower cardiovascular risk, were less anxious and depressed, were more likely to have a partner and be employed. No meaningful differences in gender, social deprivation, or physical activity were observed.

There was no gender difference in the likelihood of quitting smoking according to the results of this research. Previous research found that demographic characteristics are associated with quitting smoking; however, few such differences were identified by this research. For example, quitting smoking had no relationship with gender and being female was not predictive of quitting smoking. This was similar to the findings of numerous studies [20,26,34,46,47,48,49,50]. This is in contrast with other studies whereby some studies reported the male gender to be a strong predictor of quitting smoking [18,19], whilst other studies identified the female gender as a significant predictor of quitting smoking [23]. An important difference between this study and previous studies was the higher number of quitters in the sample and data from routine practice, which more closely resembled the range of patients in the real-world clinical setting. In addition, the finding that being older was not an important determinant of quitting smoking aligned with the results reported in a systematic review [50] by Hyland et al. [46] and by Dawood et al. [29], but was not consistent with results of previous studies [18,19,20,21]. The Prospective Registry Evaluating Outcomes After Myocardial Infarction Events and Recovery (PREMIER), with 2498 patients with MI from 19 US centers between January 2003 and June 2004, found that age and gender were not associated with quitting smoking [29]. The observational research reported here also showed that quitters were younger than smokers, indicating that older age was not a motivation to quit, however, other studies suggested that older age was another strong predictor of successful cessation [19,26,32,33,49,51].

On the other hand, a longitudinal study of the 2000 Thai adult smokers from the International Tobacco Control Southeast Asia survey, with over four years of follow-up, found that age was a strong independent predictor of quitting smoking, as older age was associated with increased success of quitting smoking [20]. Only 176 (11.8%) of the Thai survey sample (*n* = 1489) were aged >65 years, whereas 908 (27.60%) of the 3290 patients included in the analysis reported here were aged ≥65 years old, giving a more appropriate sample size for the regression analysis. The results reported by Jampaklay et al. were also consistent with those of Li et al. and Osler and Prescott in Asia and Western Europe, respectively [18,19,20]. Jampaklay et al. suggested that this might be because older people were more likely to experience health problems and thus were more motivated to quit. The percentage of smokers aged ≥55 years in the study reported by Li et al. was 23.9% in Malaysia and 31.1% in Thailand [18], compared to 63.53% in the study reported here. Osler and Prescott found that quitting smoking was associated with older age [19]; however, this study did not include individuals aged ≥60 years, while the research reported here included 1336 (40.61%) patients aged ≥60 years.

Single patients had 0.60 times lower odds of quitting smoking than patients with partners. Marital status was identified as a major predictor of quitting smoking. This was similar to the findings of the British Household Panel Survey from 1991 to 2000, in which marital status was an important sociodemographic predictor of quitting smoking [22]; the findings of Kim, who reported that being married was a significant predictor of successful smoking cessation in patients in the fourth Korea National Health and Nutrition Examination Survey (KNHANES) [23]; and the findings of West et al. who reported that smokers whose partners objected to smoking were more likely to quit [24]. Other studies also identified being married as a significant predictor of quitting smoking [22,25,29]. Although Vangeli et al. found that marital status was not related to quitting smoking, the study included the adult general population, while the research reported here included patients with CVD [50]. Moreover, these findings were consistent with the results of the large population-based sample from the 2000 National Health Interview Survey of adults in the US, which showed that quitters were more likely to be married or living with a partner [26]. The US Public Health Service clinical practice guideline also states that social support during smoking cessation increases the likelihood of quitting smoking and recommends that smokers are counselled to ask for social support from their spouse or partner(s), friends, and co-workers [52]. Quitting smoking thus seems to be influenced strongly by the social environment, and CR programs that promote smoking cessation might benefit from involving partner(s)/spouse to encourage smoking cessation. In addition, the PREMIER study found that patients who quit smoking were more likely to be married [29].

Patients with multiple comorbidities had decreased odds of quitting smoking; in contrast, low-risk patients were associated with higher odds of quitting smoking than high-risk patients. Holtrop et al. found that the presence of comorbidities in CVD patients was not associated with quitting smoking [28]. However, the results of the study might not be generalizable as it only included 136 patients (mean age 53.32 ± 9.52 years) and enrolled patients in two similar communities. Whereas in our study, 3290 CVD patients (mean age 58.23 ± 10.45 years) were included and were pooled from different communities. Numerous studies addressed the impact of individual sociodemographic characteristics or clinical measures on quitting smoking. Unlike previously cited work, a strength of our study was that our analysis accounted for known confounders such as sociodemographic characteristics, clinical measures, cardiovascular risk, and comorbidity profile. Although a Danish study found that self-rated health status was not associated with quitting smoking, it suggested that patients with high cardiovascular risk and multiple comorbidities were less likely to quit smoking [19], which is similar to the findings of the research reported here.

Patients with a higher HADS depression score had decreased odds of quitting smoking. This was in line with the finding of a meta-analysis of 42 trials by Hitsman et al., in which major depression had a moderate adverse effect on quitting smoking [53]. This finding was also in agreement with previous studies that concluded that patients who quit smoking were less likely to have depression and that depression greatly decreased the likelihood of quitting smoking [29,30].

The findings showed that identification of characteristics that predict quitting smoking among CR attendees is highly desirable, as this could help match smokers with strategies that are more likely to help them quit, identify smokers who might need more intensive treatment (who would then require referral to specialist centers), and make the most of healthcare resources. Programs designed to encourage smokers to quit might need to account for factors related to partner support as part of an existing prevention program, to encourage smokers to quit. A more wide-ranging CR offer and tailored intervention is suggested to help smokers quit, as the research findings highlight that CR programs need to prioritize patients with multiple comorbidities, high cardiovascular risk, more severe depression, and no partner(s). In future trials, it would be useful to pay increased attention to the recruitment of patients who are more representative of the broader CVD population, including those at higher risk and with major comorbidities. Clinical and research efforts should be directed towards improving the rate of smoking cessation in patients with CVD.

The strength of this study lies in the use of an observational approach based on routinely collected patient data, and the use of a large dataset taken from routine clinical practice and representing a CR intervention with a median duration of nine weeks.

Retrospective observational studies have known limitations in terms of data capture and the quality of the 230 CR programs in the UK—according to the 2019 NACR report, only 189 (82%) programs entered data electronically to the NACR. Although it can be argued that there is enough data to be representative and carry out a reliable analysis, future work should aim to achieve greater capture of available data across the UK. Although CR programs are encouraged to provide complete patient records, a proportion of patient data was expected to be missing due to non-completion of patient records. On the basis of the NACR data, 68.6% of all patients who started CR did not have a post-CR assessment recorded, which might have affected the representativeness of the research sample.

A limitation of the study was the use of self-reported data to determine smoking status, which might be subject to recall and social desirability biases. The self-reported smoking status was not validated with a biochemical marker. Some relevant factors that influenced quitting smoking were missing from the analysis, due to high levels of missing data—variables with more than 60% missing values were eliminated from the dataset (such as physical fitness) and some might not have been collected in the NACR. Some characteristics known to influence quitting smoking in the literature were not collected by the NACR, such as motivation to stop smoking, number of cigarettes per day, proportion of smokers in the household, and exposure to warning labels [18,22,27].

## 5. Conclusions

Patients with high cardiovascular risk, multiple comorbidities, no partner(s), and more severe depression were less likely to quit smoking during CR. This research highlights routine factors that determine smoking cessation outcomes and that could inform the delivery of CR to better help patients in quitting smoking.

## Figures and Tables

**Table 1:** Smoking categorization groups.

Group	Frequency (*n*)	Percent (%)
Smokers	2052	62.4
Quitters	1238	37.6
Total	3290	100

*n* = Number of patients; %, percentage of patients.

**Table 2:** Baseline characteristics of smokers and quitters participating in cardiac rehabilitation (continuous measures).

Characteristics	Group	Mean (SD)/*n*	Effect Size (Cohen’s d)
Age	Smokers	58.59 (10.49)/2052 *	0.09
	Quitters	57.63 (10.36)/1238 *	
Number of comorbidities	Smokers	1.85 (1.80)/2052 *	0.18
	Quitters	1.54 (1.58)/1238 *	
Weight	Smokers	81.63 (18.45)/1793 *	0.09
	Quitters	83.27 (18.43)/1068 *	
BMI	Smokers	28.03 (0.14)/1763	0.00
	Quitters	28.03 (0.16)/1035	
Waist	Smokers	98.72 (14.38)/918	0.01
	Quitters	98.63 (15.33)/408	
Alcohol	Smokers	9.09 (15.29)/1240	0.02
	Quitters	8.78 (14.19)/641	
HADS anxiety score	Smokers	7.31 (4.64)/1478 *	0.10
	Quitters	6.84 (4.52)/743 *	
HADS depression score	Smokers	5.96 (4.28)/1477 *	0.21
	Quitters	5.08 (4.01)/744 *	
IMD decile	Smokers	4.86 (2.90)/1585	0.01
	Quitters	4.89 (2.90)/998	

BMI—body mass index; HADS—hospital anxiety and depression scale; IMD—Index of multiple deprivation; SD—standard deviation; *n* = number of patients. * *p* < 0.05.

**Table 3:** Crosstabulation of baseline characteristics of smokers and quitters who participated in cardiac rehabilitation.

Characteristics		Original Data (%)		Effect Size (Phi/Cramér’s V)
		Smokers	Quitters	
Gender	Male	73.6	75.8	–0.02
	Female	26.4	24.2	
Ethnic group	White	77.3	77.1	0.00
	other	22.7	22.9	
Marital status	Partnered	62.1 *	73.7 *	0.12
	Single	37.9 *	26.3 *	
Work status	Employed	33.6 *	44.8 *	0.11
	Unemployed	33.6 *	27.1 *	
	Retired	32.8 *	28.1 *	
Cardiovascular risk	Low	39.5 *	49.3 *	0.10
	Moderate	38.9 *	34.6 *	
	High	21.6 *	16.2 *	
BP > 140/80 mmHg	Yes	27.1	27.6	0.01
	No	72.9	72.4	
Exercise: 150 min/week of moderate activity	Yes	30	29.8	0.00
	No	70	70.2	
Exercise: 75 min/week of vigorous activity	Yes	7.5	6.4	0.00
	No	92.5	93.6	

BP—blood pressure. * *p* < 0.05.

**Table 4:** Binary logistic regression predicting likelihood of quitting smoking among patients who attended cardiac rehabilitation.

	B	SE	Wald	df	*p*	OR	95% CI for OR	
							Lower	Upper
Age	–0.01	0.01	1.50	1.00	0.22	0.99	0.97	1.01
Marital status (single)	–0.52	0.16	10.03	1.00	0.00 *	0.60	0.43	0.82
Employment status (retired as reference)								
Employment status (employed)	0.10	0.22	0.21	1.00	0.65	1.11	0.72	1.71
Employment status (unemployed)	–0.20	0.24	0.69	1.00	0.41	0.82	0.52	1.30
Cardiovascular risk (high as reference)								
Cardiovascular risk (low)	0.54	0.22	6.10	1.00	0.01 *	1.71	1.12	2.62
Cardiovascular risk (moderate)	0.14	0.22	0.41	1.00	0.52	1.15	0.75	1.78
Comorbidities	–0.13	0.05	7.48	1.00	0.01 *	0.88	0.80	0.96
Weight	0.01	0.00	2.11	1.00	0.15	1.01	1.00	1.01
HADS anxiety score	0.03	0.02	1.57	1.00	0.21	1.03	0.98	1.08
HADS depression score	–0.06	0.03	4.16	1.00	0.04 *	0.95	0.90	1.00
Constant	–0.11	0.86	0.02	1.00	0.90	0.90		

B = unstandardized regression coefficient; CI = Confidence Interval for odds ratio; df—degrees of freedom; HADS—hospital anxiety and depression scale; OR—odds ratio; SE—standard error of the coefficient. * *p* < 0.05.

## Supplementary Materials

The following are available online at https://www.mdpi.com/2077-0383/9/8/2612/s1, Table S1: STROBE Statement—checklist of items that should be included in reports of observational studies.

## References

[1] World Health Organisation Tobacco: Fact Sheet. https://www.who.int/news-room/fact-sheets/detail/tobacco..

[2] Mons U., Muezzinler A., Gellert C., Schottker B., Abnet C.C., Bobak M., de Groot L., Freedman N.D., Jansen E., Kee F. (2015). Impact of smoking and smoking cessation on cardiovascular events and mortality among older adults: Meta-analysis of individual participant data from prospective cohort studies of the CHANCES consortium. BMJ.

[3] Wilson K., Gibson N., Willan A., Cook D. (2000). Effect of Smoking Cessation on Mortality After Myocardial Infarction. Arch. Intern. Med..

[4] Hasdai D., Garratt K.N., Grill D.E., Lerman A., Holmes D.R. (1997). Effect of Smoking Status on The Long-Term Outcome After Successful Percutaneous Coronary Revascularization. NEJM.

[5] Voors A.A., van Brussel B.L., Plokker H.W., Ernst S.M., Ernst N.M., Koomen E.M., Tijssen J.G., Vermeulen F.E. (1996). Smoking and Cardiac Events After Venous Coronary Bypass Surgery: A 15-Year Follow-up Study. Circulation.

[6] U.S. Department of Health and Human Services (2014). The Health Consequences of Smoking–50 Years of Progress: A Report of the Surgeon General.

[7] Piepoli M.F., Hoes A.W., Agewall S., Albus C., Brotons C., Catapano A.L., Cooney M.T., Corrà U., Cosyns B., Deaton C. (2016). 2016 European Guidelines on cardiovascular disease prevention in clinical practice. The Sixth Joint Task Force of the European Society of Cardiology and Other Societies on Cardiovascular Disease Prevention in Clinical Practice. Eur. Heart J..

[8] Critchley J.A., Capewell S. (2003). Mortality Risk Reduction Associated With Smoking Cessation in Patients With Coronary Heart Disease: A Systematic Review. JAMA.

[9] BACPR (2017). The BACPR Standards and Core Components for Cardiovascular Disease Prevention and Rehabilitation 2017.

[10] Gerhard-Herman M.D., Gornik H.L., Barrett C., Barshes N.R., Corriere M.A., Drachman D.E., Fleisher L.A., Fowkes F.G.R., Hamburg N.M., Kinlay S. (2017). 2016 AHA/ACC Guideline on the Management of Patients with Lower Extremity Peripheral Artery Disease: Executive Summary: A Report of the American College of Cardiology/American Heart Association Task Force on Clinical Practice Guidelines. Circ..

[11] Smith S.C., Benjamin E.J., Bonow R.O., Braun L.T., Creager M.A., Franklin B.A., Gibbons R.J., Grundy S.M., Hiratzka L.F., Jones D.W. (2011). AHA/ACCF secondary prevention and risk reduction therapy for patients with coronary and other atherosclerotic vascular disease: 2011 update. J. Am. Coll. Cardiol..

[12] Liu K., Daviglus M.L., Loria C.M., Colangelo L.A., Spring B., Moller A.C., Lloyd-Jones D.M. (2012). Healthy lifestyle through young adulthood and the presence of low cardiovascular disease risk profile in middle age: The Coronary Artery Risk Development in (Young) Adults (CARDIA) study. Circulation.

[13] NICE (2010). Cardiovascular Disease Prevention: Public Health Guideline.

[14] Balady G.J., Ades P.A., Bittner V.A., Franklin B.A., Gordon N.F., Thomas R.J., Tomaselli G.F., Yancy C.W. (2011). Referral, enrollment, and delivery of cardiac rehabilitation/secondary prevention programs at clinical centers and beyond: A presidential advisory from the american heart association. Circulation.

[15] Anderson L., Oldridge N., Thompson D.R., Zwisler A.-D., Rees K., Martin N., Taylor R.S. (2016). Exercise-Based Cardiac Rehabilitation for Coronary Heart Disease. J. Am. Coll. Cardiol..

[16] NACR (2019). The National Audit of Cardiac Rehabilitation: Quality and Outcomes Report 2019.

[17] NACR (2017). The National Audit of Cardiac Rehabilitation Annual Statistical Report 2017.

[18] Li L., Borland R., Yong H.H., Fong G.T., Bansal-travers M., Quah A.C.K., Sirirassamee B., Omar M., Zanna M.P., Fotuhi O. (2010). Predictors of smoking cessation among adult smokers in Malaysia and Thailand: Findings from the International Tobacco Control Southeast Asia survey. Nicotine Tob. Res..

[19] Osler M., Prescott E. (1998). Psychosocial, behavioural, and health determinants of successful smoking cessation: A longitudinal study of Danish adults. Tob. Control.

[20] Jampaklay A., Borland R., Yong H.H., Sirirassamee B., Fotuhi O., Fong G.T. (2015). Predictors of successful quitting among Thai adult smokers: Evidence from ITC-SEA (Thailand) survey. Int. J. Environ. Res. Public Health.

[21] Tucker J.S., Ellickson P.L., Orlando M., Klein D.J. (2005). Predictors of attempted quitting and cessation among young adult smokers. Prev. Med..

[22] Lee C., Kahende J. (2007). Factors Associated With Successful Smoking Cessation in the United States, 2000. Am. J. Public Health.

[23] Chandola T., Head J., Bartley M. (2004). Socio-demographic predictors of quitting smoking: How important are household factors?. Addiction.

[24] Kim Y.J. (2014). Predictors for Successful Smoking Cessation in Korean Adults. Asian Nurs. Res. (Korean. Soc. Nurs. Sci.).

[25] West R., McEwen A., Bolling K., Owen L. (2001). Smoking cessation and smoking patterns in the general population: A 1-year follow-up. Addiction.

[26] Broms U., Silventoinen K., Lahelma E., Koskenvuo M., Kaprio J. (2004). Smoking cessation by socioeconomic status and marital status: The contribution of smoking behavior and family background. Nicotine Tob. Res..

[27] Shang C., Chaloupka F.J., Kostova D. (2014). Who Quits? An overview of quitters in low- and middle-income countries. Nicotine Tob. Res..

[28] Holtrop J.S., Stommel M., Corser W., Holmes-Rovner M. (2009). Predictors of smoking cessation and relapse after hospitalization for acute coronary syndrome. J. Hosp. Med..

[29] Dawood N., Vaccarino V., Reid K.J., Spertus J.A., Hamid N., Parashar S. (2008). Predictors of Smoking Cessation After a Myocardial Infarction. Arch. Intern. Med..

[30] Joly B., Perriot J., D’Athis P., Chazard E., Brousse G., Quantin C. (2017). Success rates in smoking cessation: Psychological preparation plays a critical role and interacts with other factors such as psychoactive substances. PLoS ONE.

[31] von Elm E., Altman D.G., Egger M., Pocock S.J., Gøtzsche P.C., Vandenbroucke J.P. (2008). The Strengthening the Reporting of Observational Studies in Epidemiology (STROBE) statement: Guidelines for reporting observational studies. J. Clin. Epidemiol..

[32] Kim Y., Cho W.-K. (2014). Factors Associated with Successful Smoking Cessation in Korean Adult Males: Findings from a National Survey. Iran. J. Publ. Health.

[33] Hyland A., Li Q., Bauer J.E., Giovino G.A., Steger C., Cummings K.M. (2004). Predictors of cessation in a cohort of current and former smokers followed over 13 years. Nicotine Tob. Res..

[34] McMahon S.D., Jason L.A. (2000). Social support in a worksite smoking intervention: A test of theoretical models. Behav. Modif..

[35] ACPICR (2015). ACPICR Standards for Physical Activity and Exercise in the Cardiovascular Population.

[36] AACVPR (2013). Guidelines for Cardiac Rehabilitation and Secondary Prevention Programs.

[37] BACR (2006). BACR Phase IV Exercise Instructor Training Manual.

[38] NICE (2013). Hypertension in Adults (QS28).

[39] NACR (2015). The National Audit of Cardiac Rehabilitation Annual Statistical Report 2015.

[40] NICE (2010). Alcohol-Use Disorders: Prevention (PH24).

[41] Department for Communities and Local Government (2015). The English Index of Multiple Deprivation (IMD) 2015–Guidance.

[42] Department of Health and Social Care UK Physical Activity Guidelines. https://www.gov.uk/government/collections/physical-activity-guidelines..

[43] Zigmond A.S., Snaith R.P. (1983). The Hospital Anxiety and Depression Scale. Acta. Psychiatr. Scand..

[44] Cohen J. (1988). Statistical Power Analysis for the Behavioral Sciences.

[45] Field A. (2018). Discovering Statistics Using IBM SPSS Statistics.

[46] Hyland A., Borland R., Li Q., Yong H.H., McNeill A., Fong G.T., O’Connor R.J., Cummings K.M. (2006). Individual-level predictors of cessation behaviours among participants in the International Tobacco Control (ITC) Four Country Survey. Tob. Control.

[47] Westmaas J.L., Langsam K. (2005). Unaided smoking cessation and predictors of failure to quit in a community sample: Effects of gender. Addict. Behav..

[48] Babb S., Malarcher A., Schauer G., Asman K., Jamal A. (2017). Quitting Smoking Among Adults—United States, 2000–2015. Morb. Mortal. Wkly. Rep..

[49] Klimis H., Marschner S., Von Huben A., Thiagalingam A., Chow C.K. (2020). Predictors of Smoking Cessation in a Lifestyle-Focused Text-Message Support Programme Delivered to People with Coronary Heart Disease: An Analysis From the Tobacco Exercise and Diet Messages (TEXTME) Randomised Clinical Trial. Tob. Use Insights.

[50] Vangeli E., Stapleton J., Smit E.S., Borland R., West R. (2011). Predictors of attempts to stop smoking and their success in adult general population samples: A systematic review. Addiction.

[51] Holm M., Schiöler L., Andersson E., Forsberg B., Gislason T., Janson C., Jogi R., Schlünssen V., Svanes C., Torén K. (2017). Predictors of smoking cessation: A longitudinal study in a large cohort of smokers. Respir. Med..

[52] Fiore M., Jaén C., Baker T., Bailey W., Benowitz N., Curry S., Dorfman S., Froelicher E., Goldstein M., Healton C. (2009). Treating Tobacco Use and Dependence: 2008 Update. Quick Reference Guide for Clinicians.

[53] Hitsman B., Papandonatos G.D., McChargue D.E., Demott A., Herrera M.J., Spring B., Borrelli B., Niaura R. (2013). Past major depression and smoking cessation outcome: A systematic review and meta-analysis update. Addiction.

